# Inheritance of four-membrane-bound structures in the “apicoplast-minus” *Plasmodium falciparum*

**DOI:** 10.1128/msphere.00024-26

**Published:** 2026-06-29

**Authors:** Wei Xu, Ikechukwu Nwankwo, Sean T. Prigge, Hangjun Ke

**Affiliations:** 1 Department of Microbiology and Immunology, Center for Molecular Parasitology, Drexel University College of Medicine 12312, Philadelphia, Pennsylvania, USA; 2 Department of Molecular Microbiology and Immunology, Johns Hopkins Bloomberg School of Public Health 25802 , Baltimore, Maryland, USA; Weill Cornell Medicine, New York, New York, USA

**Keywords:** malaria, *Plasmodium falciparum*, apicoplast, apicoplast derivative

## Abstract

Most apicomplexan parasites contain a plastid-derived organelle called the apicoplast, which originated through secondary endosymbiosis. As a result of this evolutionary trajectory, the non-photosynthetic apicoplast is surrounded by four membranes and contains many bacterial-like, druggable targets. It is widely accepted that asexual malaria parasites (*Plasmodium falciparum*) can thrive under antibiotic treatment if supplemented with high concentrations of isopentenyl pyrophosphate (IPP, 200 µM), and these IPP-rescued parasites are thought to lack the apicoplast and its 35 kb genome but possess many vesicles. However, our findings suggest that this “apicoplast-minus” concept may warrant reconsideration. Using live-cell microscopy, we observed that apicoplast-derived vesicles closely colocalize with mitochondria in late schizonts and are faithfully segregated into merozoites during schizogony, suggesting that these vesicles are inherited from the previous cycle rather than newly synthesized. Furthermore, immuno-electron microscopy (immuno-EM) revealed that the apicoplast-minus parasites possess structures surrounded by four membranes, in addition to single-membrane-surrounded entities. The presence of four-membrane-bound structures suggests that the apicoplast has not truly disappeared in the apicoplast-minus *P. falciparum* but has remained in a distinct, diminished form. We propose that these distinct four-membrane-bound structures still retain essential biochemical and/or structural functions, which act as barriers to the complete loss of apicoplast when the parasites face antibiotic stress and IPP rescue.

**IMPORTANCE:**

The plant-like organelle named apicoplast is essential for malaria parasites and is a major antimalarial drug target. For more than a decade, scientists have believed that malaria parasites in the blood stages could dispense with the apicoplast if they were supplied with a critical metabolite made by the organelle, leading to the idea of “apicoplast-minus” parasites. Our results challenge this long-standing view. We find that even when the apicoplast is disrupted, the organelle remains in a highly reduced form. This apicoplast-derived organelle is inherited as parasites continue their life cycles, suggesting that it contains essential functions even when the organelle is disrupted. Our data reveal an unexpected level of complexity in apicoplast biology and open new doors for future identification of essential apicoplast-derived pathways that cannot be easily bypassed.

## OBSERVATION

The phylum Apicomplexa comprises a vast group of single-celled protozoa that pose a huge impact on global health and the economy by causing diseases such as malaria, toxoplasmosis, cryptosporidiosis, and coccidiosis. *Plasmodium*, the causative agent of malaria, infects 247 million people and claims 600,000 lives annually. A distinctive feature of most apicomplexan parasites is the presence of a non-photosynthetic plastid called the apicoplast, which originated from secondary endosymbiosis and is therefore surrounded by four membranes (1). The apicoplast in malaria parasites is essential for parasite survival and replication throughout their complex life cycle. Notably, antibiotics such as tetracycline (2) were used to treat malaria patients decades before the apicoplast was even identified (3, 4).

Due to its endosymbiotic origin, the apicoplast features many bacterial or plant-like pathways that synthesize critical metabolites, including fatty acids, lipoic acid, isoprenoid precursors, iron-sulfur clusters, and heme (5). During the asexual blood stage, where malaria clinical symptoms arise, the apicoplast undergoes a growth and fission cycle to divide into 16–32 copies to ensure that each progeny inherits a functional apicoplast. Despite being essential in the asexual blood stage, the apicoplast appears to undergo a reductive evolutionary trajectory in a “going, going, gone” manner, as many of its synthetic pathways—such as FASII and heme biosynthesis—are dispensable in this stage (6). Strong support for this idea came from a landmark study in 2011 (7) that demonstrated asexual *Plasmodium falciparum* could survive without an apicoplast when treated with antibiotics and rescued by sufficient amounts of IPP (200 µM), the product of the non-mevalonate/methylerythritol phosphate pathway present within the apicoplast. IPP, a five-carbon isoprene, is used for synthesizing ubiquinone and long-chain isoprenoids involved in protein prenylation and glycosylation (via dolichols). The ability of parasites to fully grow and replicate under antibiotic treatment when supplemented with IPP suggested that the sole essential function of the apicoplast in asexual blood stages is to synthesize a single molecule: IPP (7). These IPP-rescued parasites are considered to be “apicoplast-minus,” lacking the 35 kb genome and an intact organelle but possessing numerous vesicles (7).

This apicoplast-minus theory was groundbreaking, as it implied that, given access to IPP from alternative sources, malaria parasites could evolve to lose the entire organelle at least in one stage (6), mirroring the situation observed in *Cryptosporidium*, an apicomplexan parasite that has truly lost its apicoplast. Notably, *Plasmodium* and *Cryptosporidium* are estimated to have diverged approximately 420 million years ago (8). Consequently, the 2011 study suggested that a major evolutionary event, typically taking millions of years, could occur within a laboratory setting within just a matter of a few days. Since then, the concept of apicoplast-minus *P. falciparum* has been widely accepted (6, 9–15). However, here we show that the apicoplast persists in apicoplast-minus *P. falciparum* parasites, suggesting that the organelle may not be fully eliminated by antibiotic treatment and IPP rescue.

In asexual *P. falciparum*, IPP rescue can be achieved in two ways: direct addition (200 µM) or synthesis from a precursor (mevalonate, 50 µM) in an engineered parasite line called PfMev (16). Compared to direct IPP supplementation (approximately $3,500 per liter of medium), PfMev reduces the cost by several orders of magnitude (approximately $0.06 per liter of medium). Both methods are equally effective in inducing the apicoplast-minus phenotype. Importantly, PfMev is well suited for live-cell imaging, as its apicoplast is labeled by superfolder GFP (16).

**Fig 1:**
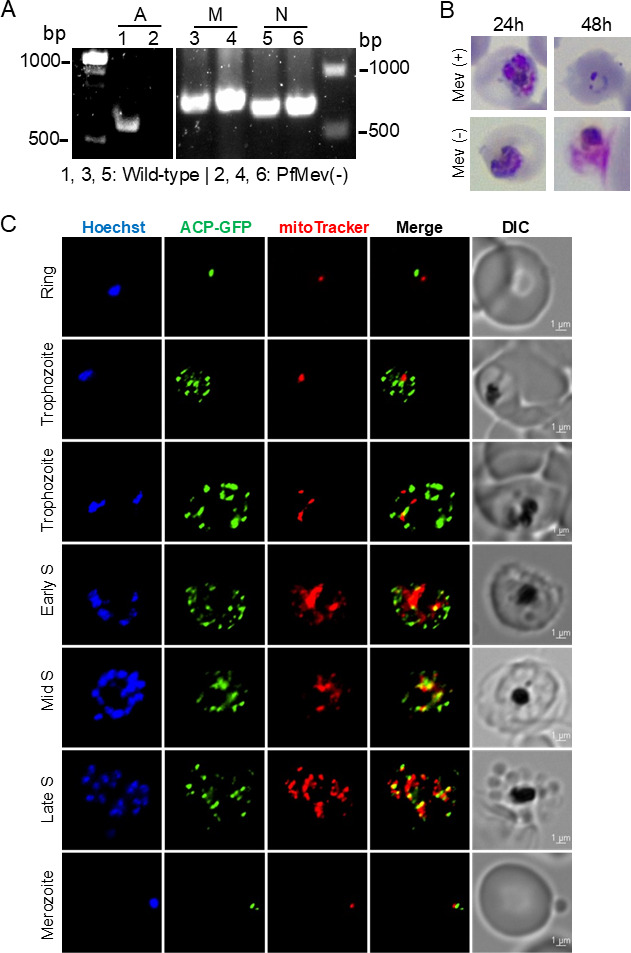
The disrupted apicoplast in the “apicoplast-minus” *P. falciparum* is inherited. (**A**) PCR verification showing the loss of apicoplast DNA in PfMev(−). The apicoplast DNA-encoded SufB (A, apicoplast), the mitochondrial DNA-encoded Cox1 (M, mitochondrion), and the nuclear gene (N, PF3D7_1037300) were amplified with PCR in genomic DNA of PfMev(−) or wild type. Primers of SufB and Cox1 were from the previous study (16). PCR reactions were carrried out by Taq polymerase as following, 25 cycles of 95C:30s, 58C:30s, and 68C:45s. (**B**) Giemsa-stained images showing PfMev’s morphology in the presence or absence of mevalonate (mev). (**C**) Live-cell imaging monitors the disrupted apicoplast in PfMev(−). Hoechst, DNA; ACP-superfolder GFP, apicoplast; MitoTracker, mitochondrion. Pearson’s correlation coefficient of green and red in 10 parasites at each stage: ring (0.440 ± 0.378); trophozoite (0.560 ± 0.127); early schizont (early S, 0.776 ± 0.034); middle schizont (mid S, 0.850 ± 0.025); late schizont (LS, 0.945 ± 0.008); and merozoite (0.915 ± 0.018). Bars, 1 µm. This experiment was repeated four times.

As shown before (16), PfMev was treated with azithromycin and 50 µM mevalonate to induce the apicoplast-minus phenotype and continuously cultured for several months. In the resulting apicoplast-minus PfMev, referred to hereafter as PfMev(−), we did not detect the SufB gene from the apicoplast genome by PCR and we confirmed no cross-contamination of any apicoplast-containing parasites, as PfMev(−) quickly died after mevalonate withdrawal (Fig. 1A and B). We stained PfMev(−) with MitoTracker and performed live-cell microscopy at different time points over the 48-hour asexual life cycle. Our observations prompt a reassessment of the prevailing apicoplast-minus model (Fig. 1C). According to this theory (7, 11), ring-stage parasites should not contain apicoplast due to the loss of the organelle in the previous cycle. In contrast, we observed a single green structure in ring-stage parasites. During the trophozoite stage, we detected numerous apicoplast-derived vesicles and the absence of an intact, tubular apicoplast, closely resembling the typical apicoplast-minus phenotype as observed in 2011 (7). Notably, these vesicles appeared to be randomly distributed throughout the parasite cytosol. However, in the schizont stage, the apicoplast-derived vesicles started to “colocalize” with the mitochondrion, suggesting a non-random association of the two organelles. This association became even more pronounced in the late schizont stage, where the apicoplast vesicles apparently “merged” with the segmented mitochondria. Upon egress, both organelles were packed into daughter cells, and they were clearly visible in the invading merozoites. While the mechanisms remain entirely unknown, our data demonstrate that the disrupted apicoplast in the apicoplast-minus parasites undergoes a process of biogenesis and distribution along with the parasite’s development and division. The inheritance of apicoplast-derived vesicles from the previous cycle was also observed in a recent study using expansion microscopy at a heightened resolution (17).

**Fig 2:**
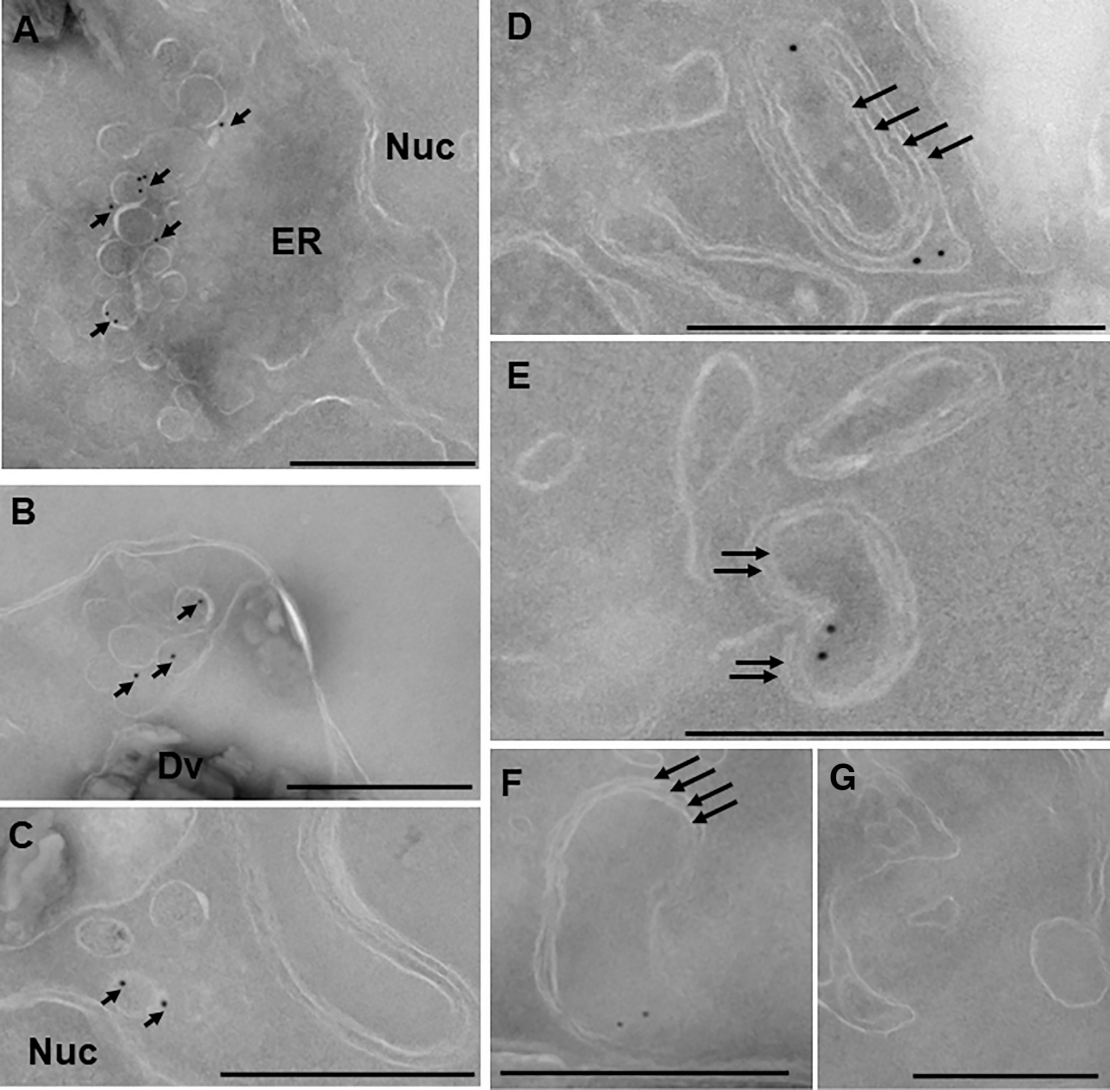
Detection of structures surrounded by four membranes in the “apicoplast-minus” parasites by immuno-EM. (**A–C**), ACP-positive structures surrounded by a single membrane (short black arrows). (**D–F**) ACP-positive structures surrounded by four membranes (long black arrows). (**G**) negative control with no primary antibody. Bars = 500 nm. Data are representative from two independent experiments that examined a total of 46 thin slices. ACP, acyl carrier protein; Dv, digestive vacuole; ER, endoplasmic reticulum; Nuc, nucleus.

To further verify the nature of the apicoplast-derived vesicles at a higher resolution, we performed immuno-EM on PfMev(−) using an antibody against acyl carrier protein (ACP) (the apicoplast marker). As shown in Fig. 2, we detected two distinct types of ACP-positive structures in the parasites. The smaller structures, approximately 50–100 nm in diameter, are circular and appear to be enclosed by a single membrane. These vesicles likely contain nuclear-encoded proteins, responsible for delivering cargoes to the apicoplast. Strikingly, the larger structures (>200 nm) are enclosed by four membranes, identifying them as the disrupted apicoplasts rather than mere vesicles. The four-membrane-architecture is the hallmark of this organelle, which has been well demonstrated with Cryo-electron tomography studies (18). Among the total of 46 thin sections from two biological replicates, we found that nearly half of the images (22/46, 47.8%) contain four-membrane-surrounded structures, and they are mostly ACP positive (91.9%), indicating an origin from the apicoplast. The presence of four-membrane-bound structures strongly suggests that the apicoplast never truly disappears in the antibiotics treated, IPP-rescued parasites. Instead, it appears to undergo an unknown transformation, becoming smaller in size and exhibiting multiple copies per parasite.

Our data suggest that the apicoplast is not lost but instead persists in a distinct, diminished form when the asexual parasites (*P. falciparum*) encounter antibiotic treatment and IPP rescue. We speculate that apicoplast derivative four-membrane-bound structures continue to serve essential biochemical and/or structural functions, acting as barriers to the complete loss of the apicoplast.

We believe that the apicoplast derivative retains core essential biochemical pathways that remain indispensable even after disruption of the organelle. Indeed, one such pathway has already been discovered: coenzyme A (CoA) biosynthesis. Deletion of the apicoplast-located dephospho-CoA kinase is lethal in PfMev despite an IPP bypass (19), likely because CoA is required by multiple subcellular compartments, including the endoplasmic reticulum (for fatty acid elongation) and the nucleus (for histone acetylation). Future verification of the structure’s proteome will likely reveal additional essential functions that cannot be bypassed by IPP supplementation. Beyond essential biochemical roles, we speculate that the derivative apicoplast may play crucial roles in organellar segregation during parasite division. A recent study suggests that the apicoplast is more closely associated with the parasite nucleus (within 50 nm) than with the mitochondrion (typically >500 nm) (20), indicating that the apicoplast, not the mitochondrion, may be responsible for leading organellar segregation. If true, this could explain why the apicoplast can never be truly lost because its absence would disrupt mitochondrial distribution and cause parasite death. Although recent studies shed some light on organellar fission in *P. falciparum* (17, 21), the mechanisms by which the daughter organelles (mitochondria and apicoplasts) segregate into merozoites remain entirely unknown. Noteworthily, PfMev(−) may become an innovative tool to study mechanisms of organellar segregation in *P. falciparum*. In summary, our discovery of four-membrane-bound structures in IPP-rescued *P. falciparum* highlights the significance of the apicoplast in malaria parasites and underscores the complexity of apicoplast function and maintenance.

Methods of parasite culture, synchronization, and immuno-EM have been described previously (22, 23). Human red blood cells were purchased from Gulf Coast Blood Center (Texas, USA). Live-cell imaging was performed with a Nikon Ti microscope, and images were processed with the Nikon NIS Elements software.
